# Association between vitamin D deficiency and multiple sclerosis- MRI significance: A scoping review

**DOI:** 10.1016/j.heliyon.2023.e15754

**Published:** 2023-04-23

**Authors:** Shorouk Hajeer, Farah Nasr, Sanaa Nabha, Marie-belle Saab, Hayat Harati, Alban Desoutter, Elie Al Ahmar, Elias Estephan

**Affiliations:** a Neuroscience Research Center, Faculty of Medical Sciences, Lebanese University, Beirut, Lebanon; b Faculty of Pedagogy, Lebanese University, Furn-El-Chebbak, Lebanon; c LBN, University Montpellier, Montpellier, France; d Faculty of Arts and Sciences, Holy Spirit University of Kaslik, Jounieh, Lebanon; e School of Engineering, Holy Spirit University of Kaslik, Jounieh, Lebanon

**Keywords:** Vitamin D, Multiple sclerosis, Magnetic resonance imaging, MRI, Disease activity

## Abstract

**Background/Objective:**

Multiple Sclerosis is a common demyelinating disease of the central nervous system. Several studies suggested a link between vitamin D deficiency and multiple sclerosis disease activity, which can be evaluated by magnetic resonance imaging. Thereby, the main objective of the following scoping review is to summarize the magnetic resonance imaging findings assessing the probable effects of vitamin D on MS disease activity.

**Methodology:**

PRISMA checklist for systematic reviews and meta-analyses was employed to structure this review. Literature was searched for observational and clinical studies tackling the given matter using several search engines including PubMed, CORE, and Embase. Data was extracted in a systematic manner, and the articles meeting the inclusion criteria were quality-assessed by Jadad scale for randomized clinical trials (RCTs) and Newcastle-Ottawa scale for observational studies.

**Results:**

A total of 35 articles were included. Twenty-one (60%) studies noted a statistically significant association between vitamin D and Multiple Sclerosis MRI-detected disease activity. MRI-detected features involved lower contrast-enhancing T1 lesions, lower hyperintense T2 lesions, and a decrease in lesions volume. On the other hand, 40% (14 articles) of the articles did not detect any significant effect of vitamin D on Multiple Sclerosis disease activity. Due to the heterogeneity of the studies involved, meta-analysis was not employed in the given review.

**Discussion/conclusion:**

There was an abundance in the number of research studies investigating the relationship between vitamin D and Multiple Sclerosis while highlighting the significant role of MRI in assessing the activity of the disease. Numerous studies found that higher serum vitamin D levels are associated with decreased new active cortical and subcortical lesions and lower lesions volume. These findings highlight the importance of imaging modalities in the various aspects of neurological diseases and encourage further research to focus on the preventive effects of vitamin D on MS patients.

## 1 Introduction

Multiple Sclerosis is one of the most common demyelinating inflammatory chronic diseases affecting the central nervous system, leading to the deterioration of myelinated neurons; thereby, causing serious neurological, cognitive, and physical defects. The underlying primary cause of Multiple Sclerosis is still not fully clear; however, research speculations have been pointing out on both genetic and environmental basis (infections, vitamins deficiency, family history, climate change, etc.) [1]. There are four main subtypes of Multiple Sclerosis including: relapsing-remitting MS (RRMS), primary progressive MS (PPMS), secondary progressive MS (SPMS), and progressive relapsing MS (PRMS) [2].

Neuroimaging is increasingly becoming a targeted tool for diagnosis, treatment, prognosis, and extensive research. Magnetic Resonance Imaging (MRI) is one modality known for its high spatial soft-tissue resolution, used as a diagnostic tool for neurological diseases, including Multiple Sclerosis. MRI is by far the most sensitive method to diagnose Multiple Sclerosis, assess the progression of its course, and monitor the treatment response [3]. Lesions are more frequently seen in areas including the periventricular region, corpus callosum, U-fibers, optic nerves, and brain stem [4].

Vitamin D is a lipid-soluble vitamin, categorized as a steroid hormone after its synthesis in the body through the liver [5]. In addition to its role in bone regulation and calcium-phosphorus homeostasis [6], more evidence is showing vitamin D's involvement in immune process and cell proliferation [7]; as vitamin D plays a major part in antigen presentation, innate and cell-mediated immune response [5]. Nevertheless, vitamin D receptors are spread almost all-over human body tissues, including major brain regions (ex: the hippocampus, substantia nigra, hypothalamus, thalamus, neocortex) [7]. It has been found that vitamin D has several essential roles within the CNS involving, but not limited to, neuronal cell differentiation and migration, synaptogenesis, neurogenesis, and neuroprotection [6]. These findings were supported by epidemiological studies suggesting that low vitamin D is associated with a higher prevalence of neurological disabilities [6] such as Alzheimer's disease (AD) [[8], [9], [10], [11]]*,* amyotrophic lateral sclerosis (ALS) [[12], [13]], and multiple sclerosis (MS) [14, 15].

Rising evidence suggests an association between Multiple Sclerosis and vitamin D. Several observational studies have found that the prevalence of Multiple Sclerosis is lower in populations which had more exposure to the sun, relating such results to the probable preventive effects of vitamin D [16].

In a cohort study investigating the effect of vitamin D deficiency on relapsing-remitting MS (RRMS) and clinically isolated syndrome MS (CIS) patients, Bäcker-Koduah et al. found that patients with higher 25(OH)D levels had a significantly lower number of T2 lesions compared to patients with lower 25(OH)D levels (60 T2 lesions VS 25 T2 lesions, respectively) [17]. Moreover, a randomized clinical trial assessing the safety and efficacy of cholecalciferol treatment in patients with RRMS revealed a significant mean reduction of new active T1 lesions and lower T1 hypointense lesions volume (p-value = 0.03) [18].

These findings suggest a possible interactive effect of vitamin D in improving the activity of Multiple Sclerosis, which will play a significant role in the preventive and therapeutic aspects of the disease. Therefore, the former justifies the need for a thorough investigation to assess the impact of vitamin D on MS patients. Several reviews have covered the given issue, but no single one has incorporated the role of magnetic resonance in assessing the effect of vitamin D on MS patients. Thus, the following scoping review covers the question of whether vitamin D would enhance Multiple Sclerosis disease activity in MS patients, with the later disease activity being assessed by MRI. Hence, the primary aim of this review is to summarize the MRI/radiological findings evaluating the effect of vitamin D on Multiple Sclerotic patients (including both observational and experimental studies).

## 2 Materials and methods

The given review followed the PRISMA checklist for systematic reviews and meta-analyses [19]. A literature search was conducted to investigate the association between vitamin D deficiency and multiple sclerosis using MRI to assess the disease's activity. No restriction date was set for the search of articles. The following review included observational and clinical studies investigating the association between vitamin D and Multiple Sclerosis, shedding light on the role of magnetic resonance imaging (MRI) in detecting the activity of the disease.

### 2.1 Search strategy and sources

Searching the literature involved selected electronic search engines, including PubMed, Cochrane, CORE, Embase, Neurology: Neuroimmunology & Neuroinflammation journal, and JAMA Neurology journal. Using advanced search methods, a combination of MeSH terms was manipulated to get more precise search results.

The primary MeSH terms used for literature search are: “magnetic resonance imaging”, “MRI”, “Multiple Sclerosis”, “MS”, “Vitamin D”, and “Vitamin D deficiency”. They were combined by Boolean operators “OR” and “AND”. The search strategy overall was as follows: (“magnetic resonance imaging” OR “MRI”) AND (“Multiple Sclerosis” OR “MS”) AND (“Vitamin D” OR “Vitamin D deficiency”).

### 2.2 Inclusion/exclusion criteria

Articles included were either observational or experimental studies, which studied the relationship between Vitamin D (independent variable) and Multiple Sclerosis (dependent variable); while considering MRI findings. Additionally, studies that recruited adult patients were only considered for review.

Studies that did not use MRI as an assessment tool for disease progression, reviews, case reports, letters to editors, protocols, and proposals were excluded. In addition, articles that were in a different language other than English were removed. Moreover, studies involving pediatrics or non-human subjects were eliminated.

### 2.3 Study selection processes

**Fig. 1:**
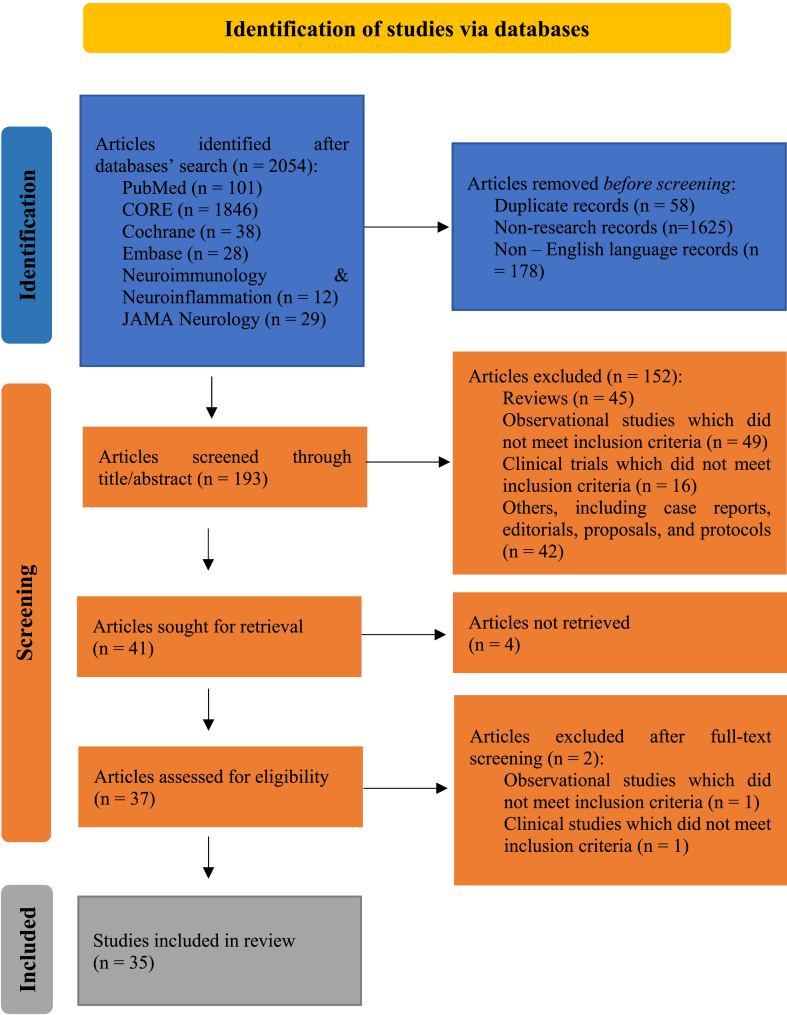
PRISMA flowchart of studies' selection.

PRISMA flowchart was employed in planning and organizing the study selection process [19]. A total of 2054 articles were extracted from different search engines. 101 articles were retrieved from PubMed, 1846 articles from CORE, 38 from Cochrane, 28 from Embase, 12 from Neurology: Neuroimmunology & Neuroinflammation journal, and 29 from JAMA Neurology journal. Fifty-eight duplicate records, 1625 non-research records, and 178 non – English language records were eliminated before screening (Fig. 1). Two main assessment steps of the articles were realized to execute this review properly. The first assessment process tackled a quick review of the title and abstract per study. Based on the latter, 152 out of the remaining 193 studies were excluded, including clinical trials and observational studies which did not meet the inclusion criteria (English language, human subjects, MRI as an assessment tool), reviews, editorials, case reports, protocols, and proposals.

Four articles could not be retrieved out of the remaining 41 articles. After full-text retrieval of the final 37 articles (Fig. 1), a second assessment step revolved around reviewing the contents of the full texts of the retrieved articles. It resulted in the removal of two more articles; one observational study which did not compare serum vitamin D levels and MRI findings directly, and a congress abstract to a peer-reviewed clinical study already included within the review. Thus, 35 articles were systematically reviewed, with proper and structured data extraction.

### 2.4 Data extraction

Two researchers, Shorouk Hajeer and Farah Nasr, were independently responsible for data screening and extraction. Depending on the study's design (experimental or observational), selected information was extracted and summarized in a table. As for observational studies, study design, sample size, objective, multiple sclerosis subtypes, and MRI findings were mainly extracted. Considering clinical studies, objective, multiple sclerosis subtype, sample size (intervention VS placebo), treatment/intervention course used, and MRI findings were reported.

### 2.5 Quality assessment

NEWCASTLE – OTTAWA Scale [20] was employed to assess observational studies. For case-control studies, one star was awarded for every item met. These items are case definition adequate with independent validation, representative series of cases, community controls, controls with no history of disease, comparability of cases and controls (and an additional factor is also given 1 star), secured records or blinded structured interviews for exposure ascertainment, same method of ascertainment for cases and controls, and same non-response rate for cases and controls.

Cohort studies were awarded as follows, with one star given for each item: true representativeness or somewhat representativeness of the average of the exposed cohort, same community selection of non-exposed cohort, secure records or structured interviews for exposure ascertainment, outcome of interest was not present at beginning of the study, comparability of cohorts (and an additional factor is also given 1 star), independent blind assessment or record linkage of outcome assessment, follow-up was enough for outcome to occur, and complete follow-up or low lost-to-follow-up rate.

Cross-sectional studies were awarded 1 star for each of the following: true representativeness or somewhat representativeness of the average of the sample, justified and satisfactory sample size, satisfactory response rate, non-validated assessment tool but is available and described, comparable outcome groups (an additional factor is also given 1 star), self-report outcome assessment, and statistical test used is described and appropriate. Articles were given two stars if they met the following criteria: validated assessment tool for exposure ascertainment and independent blind assessment or record linkage for assessing the outcome.

As for RCTs, they were assessed by Jadad scale [21] with points given as follows: 1 point was awarded for each of the given items: randomization is mentioned, randomization method is appropriate, blinding is mentioned, blinding method is appropriate, and fate of all patients in the trial is known. One point was deducted if blinding method is inappropriate, and if randomization method is inappropriate.

## 3 Results

### 3.1 Articles’ statistical description

**Fig. 2:**
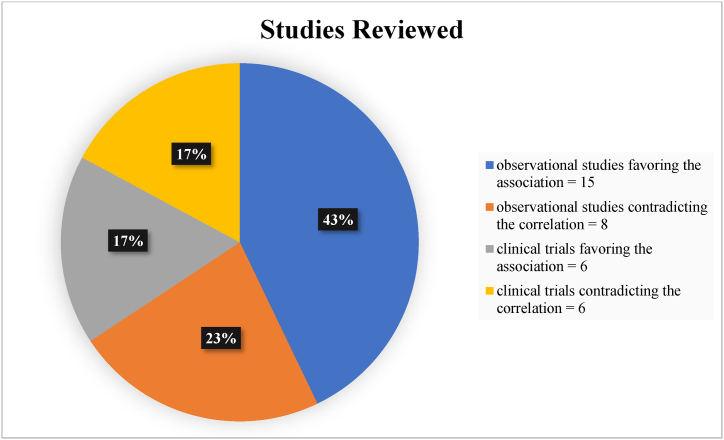
Number of studies included within each category.

Following extensive research and assessment of the findings, 35 articles were included (Fig. 1). Twenty-one studies (60%) (observational and clinical) noted a statistically significant association between vitamin D and Multiple Sclerosis disease activity as detected by MRI (Fig. 2). On the other hand, 40% (14 articles) of the total studies reviewed did not detect any significant correlation between vitamin D and Multiple Sclerosis disease activity (Fig. 2).

### 3.2 Observational studies deducing an inverse correlation between vitamin D and magnetic resonance findings among multiple sclerosis patients

Of the 23 observational studies evaluated and reviewed, 15 studies support the claim of vitamin D acting as a protective factor against the progression of Multiple Sclerosis, as assessed by MRI findings (Fig. 2). Several studies have showed an association between vitamin D and number/volume of T2 lesions as presented on magnetic resonance imaging. Bäcker-Koduah et al. significantly showed that patients with higher 25(OH)D levels had lower T2 lesions count (60 VS 25 in patients having lower 25(OH)D levels) [17]. In addition, Cree et al. deduced a short-term association between decreased serum vitamin D level and the risk of focal disease activity as seen on MRI, including T2 lesions volume [22]. Fitzgerald et al. showed that, at baseline, with every 50 nmol/l increase in 25(OH)D serum levels, a significant correlation (p-value = 0.02) of −0.11 cm^3^ in log [T2 lesion volume] is present [23]. Moreover, Linden et al. revealed that MRI scans showing new T2 lesions belonged to patients with lower 25-hydroxy-vitamin D levels (p-value = 0.030) [24].

Another subset of these observational studies presented an association between vitamin D and the number/volume of gadolinium enhancing T1 lesions. In one study, Ferre et al. prospectively followed up on patients who received fingolimod and significantly noted that those having a high serum 25[OH]D levels (≥100 nmol/l) had a lower number of gadolinium enhancing and combined unique activity lesions [25]. Hongell et al. further observed that patients who were taking fingolimod daily constituted the proportion of those free of gadolinium enhancing T1 lesions and with fewer mean lesions number [26]. Similarly, Munger et al. presented an inverse correlation showing that with every 50 nmol/l increase of 25(OH)D levels, there is a definite significant decrease in gadolinium enhancing lesions (GELs) [27]. Some studies have reported other results, including Darwish et al. who detected a significant difference in those with sufficient 25(OH)D levels to have an enhanced intracranial and total cerebellum volumes [28]. Moreover, Sistani et al. found an inverse correlation between serum vitamin D levels and cervical cord plaques (p = 0.007, r = 0.28) [29].

Other studies revealed a correlation between vitamin D and the number/volume of both T1 and T2 lesions. In a cohort studying the impact of MS patients receiving early versus late interferon beta1b (INFB-1b) treatment, results have demonstrated that patients having a serum vitamin D level of more than or equal to 50 nmol/l had a significantly lower number of isointense T1 lesions and T2 lesions than patients having a serum vitamin D level less than 50 nmol/l [30]. Loken-Amsrud et al. asserted, prior to treatment with interferon-β (IFN-β), that with each 10 nmol/l increase in 25(OH) D serum, there is a 12.7% and 11.7% decrease in new T1 gadolinium enhancing and T2 lesions respectively [31]. Furthermore, Martinelli et al. indicated that those with a more pronounced vitamin D deficiency significantly had a higher number of T2 lesions and gadolinium enhancing ones, too [32]. Mowry et al. deduced a correlation which states that with every 10 nmol/l increase in vitamin D level, there is a 15% decreased risk of developing new T2 lesions and a one-third reduced risk of having contrast-enhancing lesions [33]. Rotstein et al. found that patients who were receiving interferon-β (IFN) had a 59% hazard reduction in new gadolinium enhancing lesions per 25(OH)D tertile, compared to a 43% hazard reduction in patients receiving glatiramer acetate (GA) [34]. Nonetheless, after 12 months of interferon beta-1b treatment, the correlation persisted to a more prominent association of −0.19 cm^3^ in log [T2 lesion volume]. Wawrzyniak et al. observed that MS patients (receiving treatment) who had higher serum 25(OH)D levels had a decrease in the number of new T2 and gadolinium enhancing lesions [35].

Overall, observational studies which highlight a significant correlation between vitamin D and Multiple Sclerosis disease activity have clarified that the given correlation is an inverse one; with a higher concentration of serum vitamin D in MS patients, there are lower number and/or decrease in volume of T2 and gadolinium enhancing T1 lesions as detected on MRI.

Quality assessment was performed on these observational studies using Newcastle-Ottawa scale [20], by which a maximum of 10 stars could be earned for cross-sectional studies. On the other hand, a maximum of 9 stars could be scored for either cohort or case-control studies. The articles displayed in Table 1 had a minimum score of 8 and a maximum of 9 stars.

**Table 1:** Observational studies presenting a statistically significant relationship between vitamin D and multiple sclerosis disease activity: Study characteristics and results.

Study	Journal	NEWCASTLE – OTTAWA Score (stars)	Objective	Design/ Sample Size	Type of Multiple Sclerosis	Findings
Bäcker-Koduah et al. (2020) [17]	Frontiers in Neurology	8	To assess the relationship between hypovitaminosis D and MS disease activity	Cohort (N = 53)	Relapsing-remitting multiple sclerosis (RRMS)	Higher 25(OH)D levels were significantly associated with reduced T2 weighted lesions count (p-value = 0.03)
					Clinically isolated syndrome (CIS)	
Cree et al. (2016) [22]	Annals of Neurology	8	To characterize the long‐term disability of treated multiple sclerosis patients, and to assess the prognostic value of clinical and magnetic resonance imaging data	Prospective Cohort (517)	All subtypes	25‐OH vitamin D serum levels were inversely correlated with short‐term MS disease activity, as presented by focal MRI lesions (p-value <0.05)
Fitzgerald et al. (2015) [23]	JAMA Neurology	8	To investigate the association between 25(OH)D and disease progression in MS patients treated with interferon beta-1b	Prospective Cohort (N = 1482)	Relapsing-remitting MS	25(OH)D levels were inversely associated with the cumulative number of new active lesions (sum of new T2- and T1-enhancing lesions) (p-value = 0.01)
Linden et al. (2019) [24]	Multiple Sclerosis Journal	8	To investigate the inflammatory activity in rituximab-treated MS patients	Retrospective Cohort (272)	Relapsing MS	The number of new T2 lesions on MRI was low during rituximab treatment (p-value = 0.03)
					Primary and Secondary progressive MS	
Ferre et al. (2018) [25]	Neurological Sciences	8	To study whether baseline 25[OH]D levels could influence disease activity (during treatment with the second-line drug fingolimod (FTY))	Prospective Cohort (N = 176)	Relapsing–remitting MS (RRMS)	Patients with higher levels of 25[OH]D had a significantly lower number of gadolinium and combined unique activity (CUA) lesions (p-value <0.05)
Hongell et al. (2017) [26]	Journal of Neurology	9	To assess whether patients in the phase 3 fingolimod trials, using vitamin D supplements, have better clinical, MRI and safety outcomes	Cross sectional (N = 829)	Relapsing–remitting MS (RRMS)	Proportion of patients free of new/enlarging T2 lesions significantly favored vitamin D ‘daily users’ versus ‘non-users’ (p-value <0.05)
Munger et al. (2014) [27]	Annals of Clinical and Translational Neurology	9	To explore the mechanism that may explain clinical effects of 25(OH) D	Cross sectional (N = 465)	Clinically isolated syndrome MS	Increase in 25(OH)D levels resulted in MS activity reduction, and enhanced lesions count
Darwish et al. (2020) [28]	Journal of Steroid Biochemistry and Molecular Biology	8	To probe the effect of vitamin D changes on processing speed in MS patients, and to explore this relation with brain volume	Retrospective cohort (N = 163)	All types included	Patients with sufficient 25(OH)D levels had significant changes in intracranial (p-value = 0.001) and total cerebellum volumes (p-value = 0.012)
Sistani et al. (2019) [29]	European Journal of Translational Myology	8	To assess vitamin D status, and its seasonal fluctuations among MS patients	Prospective (N = 90)	Relapsing–remitting MS	A significant inverse correlation between serum vitamin D level and cervical spinal cord plaques (p-value = 0.007)
Fitzgerald et al. (2015) [30]	Multiple Sclerosis Journal	9	To study the association between vitamin D and the incidence of irreversible T1 hypointense lesions (permanent black holes)	Prospective cohort (N = 465)	Clinically isolated syndrome (CIS)	A significant inverse association between vitamin D and the number of T1 hypointense (p-value = 0.006) and T2 lesions (p-value = 0.056)
Loken-Amsrud et al. (2012) [31]	Neurology	8	To study the relationship between vitamin D and MS activity, while considering MRI measurements	Cohort (N = 88)	Relapsing-remitting MS	Patients with higher levels of 25(OH)D had a smaller proportion of new Gadolinium enhancing (p-value = 0.037) and T2 lesions (p-value = 0.044)
Martinelli et al. (2014) [32]	Multiple Sclerosis Journal	8	To evaluate serum 25(OH) D in patients with CIS, and examine its correlation with MS risk	Retrospective cohort (N = 100)	Clinically isolated syndromes (CIS)	Patients with low 25(OH) D levels are more likely to have enhanced lesions, and more T2 lesions (hazard ratios: 2.12 and 1.61)
Mowry et al. (2012) [33]	Ann Neurology	8	To assess if vitamin D is associated with new T2 or gadolinium enhanced lesions	Cohort (N = 469)	Relapsing multiple sclerosis MS	Higher level of 25-hydroxyvitamin D was associated with a lower risk of developing new T2 (p-value = 0.004) and contrast-enhancing lesions (p-value = 0.002)
					Clinically isolated syndromes (CIS) MS	
Rotstein et al. (2015) [34]	Neurology Neuroimmunology & Neuroinflammation	8	To check if vitamin D status correlates with disease activity in MS patients taking interferon-β (IFN), glatiramer acetate (GA), and fingolimod (FTY)	Cohort (N = 324)	Relapsing–remitting MS (RRMS)	There was a decrease in gadolinium enhancing lesions with each increase in 25(OH)D in both GA (p-value = 0.039) and IFN (p-value = 0.022) therapy subgroups
Wawrzyniak et al. (2017) [35]	Brain and Behavior	8	To study the relationship between vitamin D status, clinical and radiological outcomes	Prospective before–after study (BAF) (N = 83)	Relapsing–remitting MS	Statistically significant progression of T2‐weighted scanning sequences and contrast‐enhanced examinations (p-value <0.05)

### 3.3 Observational studies not supporting the association between MRI findings and the effect of vitamin D on MS patients

Although numerous observational studies detected a significant correlation between vitamin D on MS patients, as the disease activity was detected on MRI, several other studies are on the opposite side of the spectrum, presenting no significance (8 articles) (Table 2). Of these studies, some reported no association between vitamin D and the number/volume of T1 or T2 lesions. Meier et al. investigated the seasonal prevalence of MS while highlighting disease activity on MRI and subsequently noted that new T2 lesions/activity was 2–3 times more likely to occur between March and August (spring and summer time) [36]. Mowry et al. suggested no interaction between vitamin D and developing new T2 lesions (p-value = 0.70) [37]. In an attempt to study the relationship between sun exposure (defined by two main variables: vitamin D and latitude) and multiple sclerosis severity on patients, findings from a cohort study have proved a significant inverse association between latitude and MS disease activity, but an insignificant inverse relationship between serum vitamin D levels and the number of gadolinium enhancing T1 lesions [38]. Soilu-Hanninen detected no association between MRI BOD (burden of disease), and T2 lesions [39].

**Table 2:** Observational studies not detecting a significant agreement between vitamin D and multiple sclerosis disease activity: Study characteristics and results.

Study	Journal	NEWCASTLE – OTTAWA Score (stars)	Objective	Design/ Sample Size	Type of Multiple Sclerosis	Findings
Meier et al. (2010) [36]	Neurology	8	To probe the seasonal prevalence of MS activity, reflected by new lesions on MRI	Retrospective Cohort (N = 44)	Progressive MS	T2 activity was 2–3 times higher in the period of March–August
					Relapsing-remitting MS	
Mowry et al. (2018) [37]	Neurology	9	To determine whether BMI or vitamin D is associated with MRI measures	Cohort (N = 469)	Relapsing-remitting multiple sclerosis (RRMS) Clinically isolated syndrome (CIS)	Vitamin D levels is not significantly associated to brain volume changes (p-value = 0.6), or with new T2 lesions (p-value = 0.7)
Ostkamp et al. (2021) [38]	Proceedings of the National Academy of Sciences of the United States of America (PNAS)	9	To examine the relationship between sun exposure (latitude and vitamin D) and the severity of multiple sclerosis disease course	Cohort (N = 1519 (Two combined cohort studies)	Relapsing-remitting multiple sclerosis (RRMS) Clinically isolated syndrome (CIS)	The number of gadolinium enhancing lesions on T1 is inversely associated with latitude (p-value = 0.03), and non-significantly associated with vitamin D (p-value = 0.46)
Soilu-Hanninen et al. (2008) [39]	Neurology Neurosurgery and Psychiatry	9	To study the association between Vitamin D metabolism and MS disease activity	Case-Control (N = 46)	Relapsing-remitting multiple sclerosis (RRMS)	No association between serum 25(OH)D levels and MRI burden of disease (BOD) or T2 activity
Abbatemarco et al. (2019) [40]	Multiple sclerosis and related disorders	8	To assess the correlation between vitamin D levels and MRI features	Cohort (N = 267)	Primary progressive (PPMS)	No significant relationship between vitamin D and T1/T2 lesions volume (p-values equal to 0.59 and 0.91 respectively)
					Secondary progressive MS (SPMS)	
Lorefice et al. (2019) [41]	Multiple sclerosis and related disorders	10	To evaluate the possible effects of risk factors, including vitamin D deficiency, on brain MRI of MS patients	Cross sectional (N = 64)	Not specified	No association between serum 25(OH) D and MRI features was observed
Weinstock-Guttman et al. (2011) [42]	Neurology Neurosurgery and Psychiatry	9	To evaluate the role of vitamin D, and its metabolites in MS patients under MRI measures	Cross sectional (N = 193)	Relapsing-remitting (RRMS)	Neither total vitamin D levels nor its metabolites were significantly associated with T1/T2 lesions volume, or brain parenchymal fraction (BPF) (p-values ≥0.2)
					Secondary progressive MS (SP-MS)	
Zivadinov et al. (2013) [43]	Neurology Neurosurgery and Psychiatry	9	To assess the association of sun exposure, supplements, and environmental factors to vitamin D levels in MS patients	Cross sectional (N = 264)	Relapsing–remitting	Sun exposure was not significantly associated with T1, and T2 lesions volume findings
					Primary and Secondary progressive MS	

On the other hand, other articles highlighted the correlation between vitamin D and T1 and T2 lesions predisposition. Abbatemarco et al. concluded a non-significant association between serum 25(OH)D3 levels and T1/T2 lesion volumes (with a p-value = 0.59 and p-value = 0.91 respectively) [40]. Similar results were observed by Lorefice et al. [41] and Weinstock Guttman et al. (p-value >0.20) [42]. Zivadinov et al. investigated the effect of sun exposure on MS patients. Results indicated a statistically insignificant association between sun exposure and T1/T2 lesions, where brain changes detected on MRI could not be attributed to vitamin D status [43].

Based on the above findings, it can be noted that, in contrast to the earlier subsection, other observational studies did not find any significant association between vitamin D and Multiple Sclerosis disease activity, as evaluated by MRI. These studies either did not register a significant inverse correlation between vitamin D and MS disease activity, or it did not find a ground for any correlation between the two variables studied.

Quality assessment was performed on the above observational studies using Newcastle-Ottawa scale [20], by which a maximum of 10 stars could be earned for cross-sectional studies, and a maximum of 9 stars could be scored for either cohort or case-control studies. The scores of the above articles ranged between 8 and 10 stars (Table 2).

### 3.4 Observational studies’ statistics

**Fig. 3:**
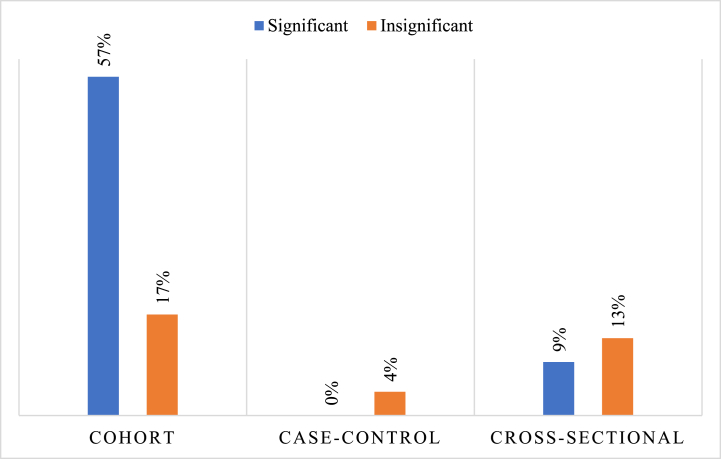
Percentage of studies showing significant association between vitamin D status and MRI-detected disease activity VS studies detecting insignificant association, in terms of the design of the observational studies included (23 total observational studies).

Most observational studies are composed of cohort studies with a percentage of 73% (17 out of 23 articles) (Fig. 3). On the contrary, case-control studies represented only 4% (1 out of 23 articles). The latter gives an insight that an abundant number of studies done in literature follow up on patients receiving vitamin D supplements/treatment to better test the probable preventive effects of vitamin D on the activity of Multiple Sclerosis.

### 3.5 Randomized control trials (RCTs) that detected a statistically significant association between vitamin D, and multiple sclerotic activity as evaluated on MRI

Of the articles reviewed, 12 out of 35 studies constituted an RCT; six studies confirmed a significant relationship. Multiple studies registered a significant association between vitamin D and incidence of T1 and/or T2 lesions. Aivo et al. noted that MS patients who received cholecalciferol had a lower number of gadolinium enhancing lesions (p-value = 0.027) [44]. Ascherio et al. asserted that higher levels of serum 25(OH)D were associated with a lower occurrence of new active lesions and a decrease in T2 lesions volume (as those who had a 50 nmol/l increase in serum 25(OH)D showed a 57% decrease in new active lesions incidence, and a 20% of that in T2 lesions volume for the first year) [14]. Camu et al. assigned cholecalciferol to the treated group in an RCT, and they found a significant mean reduction of new T1 lesions and a decrease in T1 hypointense lesions volume (p-value = 0.03) [18]. Similarly, Soilu-Hanninen probed the efficacy of vitamin D3 as an add-on therapy for MS patients who were previously treated with interferon beta-1b. Patients in the treatment group had a significantly lower number of T1 enhancing lesions (p-value = 0.004), and a non-significantly lower number of new T2 lesions than the placebo group [45].

Derakhshandi et al. observed a significant lower incident rate of new enhancing (p-value = 0.002), new T2 (p-value = 0.001), corpus callosal (p-value = 0.005), and other cortical lesions in the treated subgroup who were administered 50,000 IU of vitamin D3 [46]. Hupperts et al. results further revealed a 32% reduction in combined unique active (CUA) lesions in the group treated with high-dose vitamin D_3_ and in the total number of T2 lesions (p-value = 0.035) [47].

As stated above, some randomized clinical trials have shown that with the administration of vitamin D supplementations, there is a decrease in the number/volume of T2, gadolinium enhancing T1, and active lesions.

Randomized clinical trial studies underwent quality evaluation by Jadad scale [21], by which a maximum score of 5 points could be given to a clinical study. The RCT articles provided in Table 3 scored between 2 and 5 points.

**Table 3:** Clinical trials showing a significant association between vitamin D and multiple sclerosis disease activity: Study characteristics and results.

Study	Journal	Jadad et al. Score (points)	Objective	Sample Size (intervention/placebo)	Type of Multiple Sclerosis	Intervention	Findings
Ascherio et al. (2014) [14]	JAMA Neurology	2	To determine whether 25 hydroxyvitamin D (25(OH)D), predicts disease activity and prognosis in CIS patients	292/176	Clinically isolated syndrome CIS	Interferon beta-1b (IFNB-1b 250 μg)	Higher levels of 25(OH)D were associated with less T2 lesion volume (p-value <0.001), and decreased rate of occurrence of new active lesions (p-value <0.001)
Camu et al. (2019) [18]	Neurology Neuroimmunology & Neuroinflammation	5	To study the safety and efficacy of cholecalciferol in patients with RRMS	45/45	Relapsing-remitting MS (RRMS)	Cholecalciferol 100,000 IU	Cholecalciferol led to less new hypointense T1 lesions (p-value = 0.025), and to lower lesions volume (p-value = 0.031)
Aivo et al. (2012) [44]	Multiple Sclerosis International	4	To study safety and efficacy of interferon beta-1b therapy in MS patients	32/30	Relapsing-remitting MS	20 mg of cholecalciferol (Corresponding to 20000 IU of vitamin D3)	Statistically significant reduction in the number of T1 enhancing lesions (*P*-value = 0 .027), and less new T2 brain MRI lesions (*P*-value = 0 .132)
Soilu-Hänninen et al. (2012) [45]	Journal of Neurology, Neurosurgery & Psychiatry	5	To probe the efficacy of using vitamin D3 as an add-on treatment on MS patients who were previously treated with interferon beta-1b	34/32	Relapsing-remitting MS	Dekristol (vitamin D3)	Treated group were presented with a statistically significant lower number of T1 enhancing lesions in comparison to placebo group (p-value = 0.004)
Derakhshandi et al. (2013) [46]	Acta Neurologica Belgica	5	To evaluate preventive effects of vitamin D3 on conversion of Optic Neuritis to MS	13/11	Not specified (with optic Neuritis)	50,000 IU of vitamin D3	The incidence rates of, new gadolinium enhanced (p-value = 0.002), and new T2 lesions were significantly lower in the treated group (p-value = 0.001)
Hupperts et al. (2019) [47]	Neurology	5	To assess efficacy and safety of vitamin D3 in patients with RRMS	113/116	Relapsing-Remitting Multiple Sclerosis (RRMS)	High-dose vitamin D_3_14,007 IU/d	High-dose vitamin D3 was associated with decreased number of combined unique active (CUA) lesions (p-value = 0.0045), and reduced mean percentage change in total volume of T2 lesions (p-value = 0.035)

### 3.6 Randomized clinical trials showing no significant association between MS activity and vitamin D as evaluated by MRI

Contrary to the previous results, several RCTs did not confirm an association after the applied intervention (Table 4). Dorr et al. registered a non-notable difference in total T2 lesions count (p-value = 0.15), T2 lesions volume (p-value = 0.98), and the number of new enhancing lesions (p-value = 0.09), while comparing treated groups to baseline data [48]. Mosayebi et al. demonstrated no difference in the number of enhancing lesions among treated (with vitamin D3) and control groups [49]. Studying the effect of atorvastatin on MS patients, Mowry et al. results suggested no improvement, as the number of new T2 lesions and that of enhancing T1 lesions were indifferent between the treatment and placebo subgroups (statistically insignificant) [50]. O'Connell et al. did not detect any difference in the number of new T2 lesions or the proportion of patients showing new activity on MRI between treated patients with vitamin D3 and placebo (comparing to baseline) [51]. Stein et al. assessed the probable effects of high dose vitamin D2 versus low dose and observed non-significant changes at the level of new gadolinium enhancing lesions (p-value = 0.7) and T2 lesions volume (p-value = 0.6) [52]. Moreover, Wingerchuk et al. found that 29% of patients still showed enhancing lesions after treatment with oral calcitriol (out of the 33% showing enhancing lesions at baseline) [53].

**Table 4:** Clinical trials not finding a significant association between vitamin D and multiple sclerosis disease activity: Study characteristics and results.

Study	Journal	Jadad et al. Score (points)	Short Description	Sample Size (intervention /placebo)	Type of Multiple Sclerosis	Intervention	Findings
Dorr et al. (2020) [48]	Multiple Sclerosis Journal	5	To compare effects between high and low doses of cholecalciferol on patients with RRMS or CIS	21/17	Relapsing–remitting MS	High dose (20,400 IU) versus low dose (400 IU) cholecalciferol	Number of new T2-weighted hyperintense lesions, T2 lesions volume, number of contrast-enhancing lesions, and brain atrophy did not differ between both treatment arms (p-value >0.005)
					Clinically isolated syndrome		
Mosayebi et al. (2011) [49]	Immunological Investigation	4	To study the effect of short-term vitamin D therapy on MS patients	26/33	Relapsing–remitting MS	300,000 IU vitamin D3	No significant difference between the treatment and the control groups in the number of gadolinium enhancing lesions
Mowry et al. (2016) [50]	European Journal of Neurology	4	To investigate the association between vitamin D levels and brain activity and volume in CIS	39/26	Clinically isolated syndrome (CIS)	Atorvastatin 80 mg	25-hydroxyvitamin D levels did not appear to be associated with the number of new T2 lesions (p-value = 0.35), or gadolinium enhancing lesions (p-value = 0.77)
O'Connell et al. (2017) [51]	Multiple Sclerosis Journal	4	To assess the immunological effects of vitamin D3 in clinically isolated syndrome and healthy control	23 (taken 10000 IU)/23 (taken 5000 IU)/18 (taken placebo)	Clinically isolated syndrome (CIS)	5000 IU or 10,000 IU vitamin D3 (Vigantol oil)	No significant differences were noted in the number of new T2 and gadolinium enhancing lesions between groups
Stein et al. (2011) [52]	Neurology	4	To study the effects of high-dose vitamin D2 in MS patients	11/12	Relapsing-remitting MS (RRMS)	High-dose vitamin D2, 6000 IU **versus** low dose	No significant difference between treatment groups in the number of new gadolinium enhancing lesions (p-value = 0.75), nor in the volume of lesions on T2-weighted imaging (p-value = 0.45)
Wingerchuk et al. (2005) [53]	Neurology Neurosurgery & Psychiatry	1	To probe the safety and tolerability of oral calcitriol in an open label study	15	Relapsing–remitting MS	Oral calcitriol (target dose: 2.5 mg/d)	Brain MRI showed enhancing lesions in 9 patients, over the course of the treatment

In contrast to the preceding subsection, other experimental studies did not find a significant association between vitamin D and Multiple Sclerosis disease activity. Despite these studies supplementing MS patients with vitamin D dosages, documented results either did not show an improvement within these patients or the differences found between experimental and control groups were not statistically significant.

Experimental studies underwent quality evaluation by Jadad scale [21], where a maximum score of 5 points could be given to a clinical study. The RCT articles provided in Table 4 scored between 1 and 5 points.

## 4 Discussion

The current scoping review is the first one aimed at revising the literature in what concerns the role of magnetic resonance imaging in assessing the probable effects of vitamin D on Multiple Sclerotic patients, proving the significance of MRI in detecting the progression/activity of the disease. Searching the literature, observational and clinical studies were included, and there was no restriction on the timeline. Thirty-five articles were evaluated for their quality as observational studies by Newcastle-Ottawa scale [20] and as experimental studies by Jadad scale [21], separately depending on study design. 60% of the obtained/evaluated studies showed a significant relationship between vitamin D and Multiple Sclerosis disease activity under the evaluation of MRI, which presented several imaging features relating to the association. The latter included: fewer new cortical and subcortical lesions (active T2 lesions and contrast-enhancing lesions on T1), and lower overall lesions volume. Contrary to the former findings, 40% of the studies (14 articles) were on the opposite side of the spectrum. Despite patients within these studies having higher serum vitamin D levels and/or being treated with vitamin D supplements, no statistically significant results were noted.

There is an abundance of the studies probing the relationship between vitamin D and Multiple Sclerosis MRI-detected disease activity, which is a clear strength embodied within this review. Moreover, cohort studies made up a great percentage of the observational studies executed (17 out of 23 articles (73%)), indicating that the most observational studies focus on following up on a prior intervention given to MS patients. Despite the high heterogeneity of the studies included (in design, methods, etc.), there are common ground findings which could be concluded from the various investigations and results. The most frequent ones are the number of new hyperintense T2 lesions, new T1 contrast-enhancing lesions, and lesions volume. Whether the study carried out suggested a significant correlation or not, the former common findings were highlighted.

**Fig. 4:**
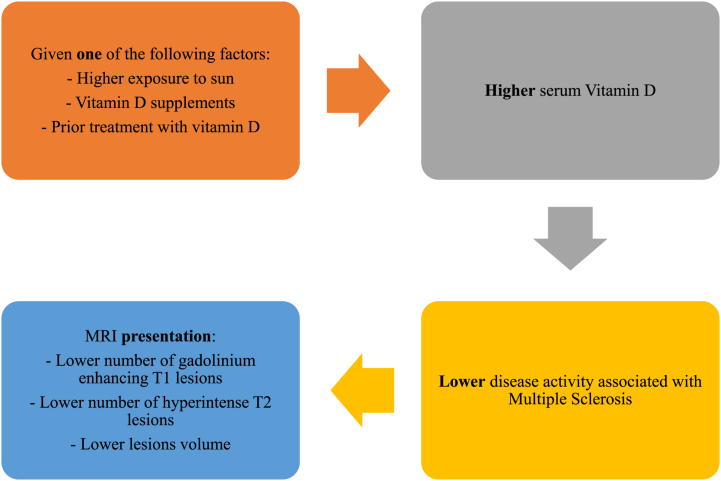
Relationship between vitamin D and Multiple sclerosis.

Building up on the previous results, our review has successfully covered its main objective to summarize the MRI findings, evaluating the correlation between vitamin D and Multiple Sclerosis disease activity, as clearly viewed in Fig. 4. There was a definite tendency toward a positive impact upheld by vitamin D on MS patients, as revealed by the enhanced disease activity evaluated by magnetic resonance imaging. It is worth mentioning that some of the studies which did not register a significant association showed improvement in disease activity (though insignificant) after treatment with vitamin D [52]. Thus, our review infers an association between vitamin D and Multiple Sclerosis disease activity whilst highlighting the role of MRI in detecting the correlation, which is considered the biggest contribution of our review, hence shedding light on the growing significance of imaging modalities in the various fields of diagnosis, prognosis, treatment, and follow-up.

This significant association could be explained by the fact that there are numerous vitamin D receptors spread all over the different regions of the brain, including both cortical and subcortical areas [7]. Nonetheless, vitamin D has been clinically proven to have a role in neuroinflammation [54]. Research has identified that the processed and resultant vitamin D3, within the central nervous system, fosters the anti-inflammatory phenotypes expressed by B-cells, T-cells, and macrophages [54]. Additionally, vitamin D plays a crucial role in promoting myelination [55]; studies have revealed that, while regulating oligodendrocyte precursor cells (OPC), vitamin D is capable of decreasing the rate of demyelination, while stimulating remyelination in the central nervous system [55]. Consequently, such physiological and functional presence of vitamin D in the central nervous system can somehow give insight into the association between vitamin D deficiency and various neurological-immunity defects [6].

Being a scoping review, limitations are not far from reach. Studies included in the current review are presented with high heterogeneity in what concerns several aspects. These aspects involve, but are not limited to, study design (observational or experimental), methodology carried out (interventional or prospective/retrospective study), underlying exposure (sun exposure, vitamin D supplements, etc.), and targeted population (various subtypes of Multiple Sclerosis). Thus, results and findings could not be combined and assessed with a single statistical test (size effect calculation). Articles were restricted by language. Furthermore, there was no single search engine to have involved all articles published which tackled the problem investigated herein, thus creating a database bias. Nevertheless, data provided in the extraction results were limited to a selected set of information.

Although there is significant deviance towards a clear association between vitamin D and subsequent MS disease progressive course, contradicting results exist among the articles involved in this review. To resolve such issue, our recommendations for further research lie in taking into account the different subtypes of the disease, extending of the follow-up period, a larger and more representative number of participants, decrease of confounding factors among study subjects (age, gender, accompanying diseases, etc.), and focus on the type/dose of administered vitamin D. Additionally, we propose delving more into the cellular/molecular aspect, to better investigate the probable mechanism underlying the effect of vitamin D on the central nervous system functionality and structure.

## 5 Conclusion

The conducted scoping review presented, qualitatively speaking, a tendency towards a significant association between vitamin D status and Multiple Sclerosis disease activity, as it was assessed by the number of hyperintense T2 and gadolinium enhancing T1 lesions and their given volume on magnetic resonance imaging. Such findings indeed call for more in-depth research on the given topic, for it suggests a probable effective and preventive method to lessen the drastic progression of the disease somehow. Thereby, administering vitamin D to MS patients could promise an improved walk into patients’ care, prognosis, and general life expectancy.

## Author contribution statement

All authors listed have significantly contributed to the development and the writing of this article.

## Data availability statement

Data associated with this study has been uploaded on figshare. The DOI is 10.6084/m9.figshare.22550575.

## Declaration of competing interest

The authors declare that they have no known competing financial interests or personal relationships that could have appeared to influence the work reported in this paper.
